# The effects of aerobic exercise on body composition in overweight and obese patients with gout: a randomized, open-labeled, controlled trial

**DOI:** 10.1186/s13063-022-06695-x

**Published:** 2022-09-05

**Authors:** Ertao Jia, Haiqiong Zhu, Hongling Geng, Ruilin Liu, Xueqian Wo, Yaochi Zeng, Wukai Ma, Xueming Yao, Zhiying Zhan, Jianyong Zhang

**Affiliations:** 1 The Department of Rheumatology, Shenzhen Traditional Chinese Medicine Hospital, Shenzhen, China; 2 The Department of Rheumatology, the Fourth Clinical Medical College of Guangzhou University of Chinese Medicine, No.1, Fuhua Road, Futian District, Shenzhen, 518033 Guangdong China; 3 Shenzhen Traditional Chinese Medicine Hospital Affiliated to Nanjing University of Chinese Medicine, Shenzhen, China; 4 The Department of Gynecology, Guangdong Provincial Hospital of Chinese Medicine, The Second Affiliated Hospital of Guangzhou University of Chinese Medicine, Guangzhou, China; 5 The Department of Cardiology, Shenzhen Traditional Chinese Medicine Hospital, Shenzhen, China; 6 The Department of Nutrition, Shenzhen Traditional Chinese Medicine Hospital, Shenzhen, China; 7 The Department of Rheumatology, the Second Affiliated Hospital of Guizhou University of Traditional Chinese Medicine, Guiyang, China; 8 The Department of Epidemiology and Health Statistics, Fujian Provincial Key Laboratory of Environment factors and Caner, School of Public Health, Fujian Medical University, Fuzhou, China

**Keywords:** Gout, Body composition, Treadmill exercise test, Aerobic exercise, Randomized controlled trial

## Abstract

**Background:**

Overweight and obesity are typical risk factors for the increased prevalence and incidence of gout. The existing guidelines unequivocally indicated that exercise is highly advantageous for patients with gout. Nevertheless, there is still a lack of specific guidance and clinical evidence. The effects of exercise on improving gout, and the optimal frequency, timing, and types of exercise have not been fully clarified. The present trial aims to determine the effects of a specific aerobic exercise program on body composition in overweight and obese patients with gout.

**Methods:**

In this randomized, open-labeled, controlled trial, a total of 60 overweight and obese patients with gout [body mass index (BMI) ≥ 24 kg/m^2^; age,18–55 years old] are equally randomized (1:1) into two groups (*n* = 30): moderate-intensity aerobic exercise group (MIAEG), heart rate reserve (HRR) = [(HRmax-HRrest) × 60% intensity] + HRrest, and control group (CG). The moderate-intensity aerobic exercise training program will be conducted for 30–40 min/session and 3 days/week for 12 weeks. Participants in the CG will be asked to avoid making changes in their exercise habits. There will be no limitation in the type of exercise. The primary outcome is the number of patients whose body fat is reduced after 12 weeks. The secondary outcomes include the changes in BMI, waist-to-hip ratio (WHR), insulin resistance index (IRI), serum uric acid (sUA), serum creatinine (SCr), estimated glomerular filtration rate (eGFR), triglycerides (TG), total cholesterol (TC), low-density lipoprotein cholesterol (LDL-C), high-density lipoprotein cholesterol (HDL-C), hepatic steatosis, and adverse effects after 12 weeks. One-way analysis of variance (ANOVA) will be used to compare the mean values of normally distributed variables between MIAEG and GC.

**Discussion:**

The effect and optimal frequency of exercise for improving the status of overweight and obese patients with gout have not yet been determined. We design a 12-week randomized controlled trial and evaluate the effects of individualized aerobic exercise program on patients with gout. The results may assist such patients with a personalized scientific exercise program based on the disease status and motor abilities, so that patients are prone to exercise under the condition of low risk and achieve the greatest benefits.

**Trial registration:**

ChiCTR2200062153. Registered on July 25, 2022, with ChiCTR. http://www.chictr.org.cn/

## Administrative information

**Table:** 

Title {1}	The effects of aerobic exercise on body composition in overweight and obese patients with gout: A randomized, open-labelled, controlled trial
Trial registration {2a and 2b}.	ChiCTR2200062153. Registered on July 25, 2022, with ChiCTR. http://www.chictr.org.cn/
Protocol version {3}	16 May 2022, V.20220408
Funding {4}	National Natural Science Foundation of China (82174290) are not involved in the design of the study and collection, analysis, and interpretation of data and in writing the manuscript.
Author details {5a}	Ertao Jia, Jianyong Zhang. The Department of Rheumatology, Shenzhen Traditional Chinese Medicine Hospital,Shenzhen, China. The Department of Rheumatology, the Fourth Clinical Medical College of Guangzhou University of Chinese Medicine,Shenzhen, China. Haiqiong Zhu, Ruilin Liu. Shenzhen Traditional Chinese Medicine Hospital Affiliated to Nanjing University of Chinese Medicine, Shenzhen, China. Hongling Geng. The Department of Gynecology, Guangdong Provincial Hospital of Chinese Medicine, the Second Affiliated Hospital of Guangzhou University of Chinese Medicine, Guangzhou, China. Xueqian Wo. The Department of Cardiology, Shenzhen Traditional Chinese Medicine Hospital, Shenzhen, China. Yaochi Zeng. The Department of Nutrition, Shenzhen Traditional Chinese Medicine Hospital, Shenzhen, China. Wukai Ma, Xueming Yao. The Department of Rheumatology, the Second Affiliated Hospital of Guizhou University of Traditional Chinese Medicine, Guiyang, China. Zhiying Zhan. The Department of Epidemiology and Health Statistics, Fujian Provincial Key Laboratory of Environment factors and Caner, School of Public Health, Fujian Medical University, Fuzhou, China.
Name and contact information for the trial sponsor {5b}	Shenzhen Traditional Chinese Medicine Hospital. No.1, Fuhua Road, Futian District, Shenzhen, Guangdong, China, 518033
Role of sponsor {5c}	The sponsor played no part in study design; collection, management, analysis, and interpretation of data; writing of the report; and the decision to submit the report for publication.

## Introduction

### Background and rationale {6a}

Gout is mainly a metabolic syndrome caused by purine metabolism disorder, and uric acid (UA) excretion disorder and (or) increased UA production is one of the leading causes of purine metabolism disorder. A meta-analysis reported that the overall prevalence of gout in China was 1.1%, and its prevalence among men (1.5%) was higher than that among women (0.9%). Besides, gout has become the second most common metabolic disease after diabetes [1]. In the recent 20 years, improvement in living standards and changes in lifestyle and diet plan have led to the remarkably increase of the incidence of gout. The age of onset of gout has gradually become younger. There is a consensus that hyperlipidemia, hypertension, and type 2 diabetes are more prevalently detected in patients with gout [2]. These diseases all belong to the subgroups of metabolic syndrome. Gout and the subgroups of metabolic syndrome are reciprocal causation, influence each other, exacerbate the illness, and even substantially increase the risk of cardiovascular disease.

At present, the gout is known to be associated with purine metabolism disorder, serum UA (sUA) metabolism disorder, diet and lifestyle, and is affected by genetic and environmental factors. Overweight and obesity were reported as typical risk factors for the increased prevalence and incidence of gout [3]. In a Mendelian randomization study, a higher body mass index (BMI) was positively correlated with the risk of gout and sUA concentration. Each standard deviation (about 4.6 kg/m^2^) increase in BMI was associated with an odds ratio (OR) of 2.24 for gout and with a 0.30 mg/dl increase in sUA level [4]. Intervention with bariatric surgery, which prevented gout and hyperuricemia in obese subjects, reduced the incidence of gout by 40% over 26 years of follow-up [5].

Moderate-intensity and regular physical exercise can significantly contribute to the long-term survival of patients with gout. A study demonstrated that light- and moderate-intensity exercises cause anti-inflammatory effects to varying degrees. Nuclear factor-κB (NF-κB) activity, synovial infiltration by macrophages and neutrophils, Toll-like receptor 2 (TLR2) expression level in peripheral neutrophils, and chemokine ligand 1 (CXCL1) expression level in serum were downregulated after light- and moderate-intensity exercises [6]. Additionally, aerobic exercise decreases sUA level by accelerating ATP turnover rate (indicated by the increase in serum phosphorus level and the decrease in serum levels of ATP, adenosine, and hypoxanthine) [7], whereas exercise is a double-edged sword with dual effects on the body. It is noteworthy that the body would accelerate the degradation of purine nucleotide and generate a large amount of lactic acid during anaerobic exercise, and the accumulated lactic acid would affect the excretion of UA by the kidney, associating with the increase of UA concentration and the likelihood of gout flares [8]. Vigorous exercise greatly increases the serum antioxidant capacity and also causes a short-term increase in UA level [9]. Hence, the effects of intensity and frequency of the certain modality of exercise, along with duration of the exercise on disease are various. Aerobic exercise such as brisk walking, jogging, cycling, and Tai chi is often a preferred mode of exercise due to its simplicity and practicality than engage in vigorous exercise.

The guidelines unequivocally indicated that exercise is one of the non-pharmacological measures for patients with gout [10–12]. Nevertheless, there is still a lack of specific guidance and clinical evidence, and the effects of exercise on improving gout and the optimal frequency, timing, and type of exercise have not been fully clarified. Gout is a continuous, chronic pathological process that requires long-term and even lifetime health care, while chronic disease management of gout is still suboptimal in several countries. The primary reason for patients' insufficient exercise participation is that the disease itself has a noticeable influence on the exercise capacity, resulting in loss of patients’ confidence in exercise. A vicious circle is eventually developed. Thus, selection of a valid and adherence of exercise program has attracted rheumatologists’ attention. Moderate-intensity aerobic exercise does not cause remarkable fluctuations in lactic acid concentration and affects UA excretion in patients with a lower risk of gout. Treadmill exercise test (TET) is a scientific assessment of exercise tolerance based on metabolic equivalent (METs). The target heart rate and exercise speed of patients with gout will be assessed by the Heart rate reserve method and Bruce protocol, respectively [13]. The aim is to provide data support for the development of personalized exercise prescriptions as well as provides a bottom line for exercise risks.

### Objectives {7}

The present trial aims to determine the effects of a specific aerobic exercise program on body composition in overweight and obese patients with gout.

### Trial design {8}

**Fig. 1:**
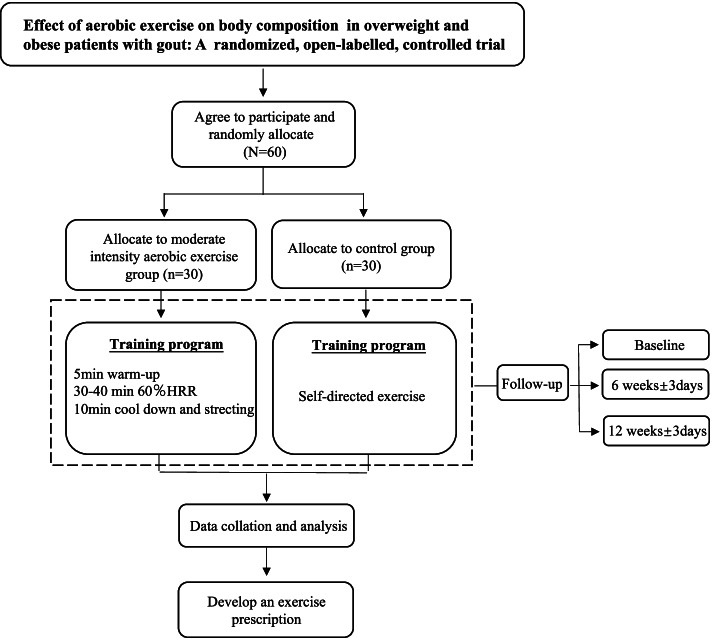
Trial flow and study design

This is a randomized, open-label, controlled trial over 12 weeks. A total of 60 patients with gout who meet the study criteria are randomized (1:1) to the moderate-intensity aerobic exercise group and control group (Fig. 1). The trial framework is exploratory.

## Methods: participants, interventions, and outcomes

### Study setting {9}

The present study will be completed at Shenzhen Traditional Chinese Medicine Hospital, People’s Hospital of Longhua District Shenzhen, and the Second Affiliated Hospital of Guizhou University of Traditional Chinese Medicine from May 16, 2022, to May 16, 2024. Written informed consent is obtained from all participants prior to the commencement of the study. All enrolled participants will be fully informed about the objective, methods, and possible risks of the study.

### Eligibility criteria {10}

#### Inclusion criteria

Patients who meet the following criteria will be included:

1. Male or female patients aging 18–55 years old;
2. BMI ≥ 24 kg/m^2^;
3. The diagnosis meets the 2015 European League Against Rheumatism (EULAR)/American College of Rheumatology (ACR) criteria for gout;
4. The absence of gout flares in the last 2 weeks;
5. Patients with complete clinical data who voluntarily participate in the study and sign the informed consent form.

#### Exclusion criteria

The following exclusion criteria will be applied:

1. Patients without normal locomotor activity due to joint deformity;
2. Secondary hyperuricemia caused by nephropathy, hematologic abnormalities, certain medications, tumor radiotherapy or chemotherapy, organ transplantation, etc.;
3. Consumption of drugs that increase sUA concentration, such as proton pump inhibitors and H2 receptor antagonists;
4. Consumption of drugs that promote excretion of UA (e.g., benzbromarone);
5. Severe cardiac diseases (e.g., acute myocardial infarction within 3–5 days, unstable angina, uncontrolled arrhythmias that was accompanied by severe hemodynamic symptoms, acute pericarditis and myocarditis, severe hypertension (> 180/110 mmHg)/hypotension (< 85/50 mmHg), aortic dissection, aortic stenosis, hypertrophic obstructive cardiomyopathy, cardiac failure, moderate-to-severe left main coronary artery, unsuccessful control of hypertension, cardiac dysfunction, bradyarrhythmias (< 45 beats per minute (bpm)) or tachyarrhythmias (> 125bpm);
6. Severe brain diseases (e.g., stroke and transient ischemic attack);
7. Severe pulmonary diseases (e.g., acute pneumonitis, acute pulmonary embolism, chronic obstructive pulmonary disease, and pulmonary hypertension);
8. Severe lower limb diseases (e.g., upper extremity deep vein thrombosis);
9. Systemic diseases (e.g., severe anemia, high fever, electrolyte disturbances and digitalis toxicity);
10. Other diseases that may be aggravated by exercise (e.g., multiple infectious diseases, renal insufficiency, hyperthyroidism);
11. Complication of gout with mental disorders;
12. Patients who have been involved in other clinical investigations within the first 3 months of enrolment.

### Who will take informed consent? {26a}

The subjects in this study will be recruited from outpatient and inpatient wards by placing advertisements on social media platforms of the hospital departments and distributing posters in public areas of the hospital with details of the study and contact information. Patients who meet the criteria will be invited to participate in the study, and they will be provided with the details of the RCT protocol. All subjects will be required to sign an informed consent by the clinicians.

### Additional consent provisions for collection and use of participant data and biological specimens {26b}

We will request consent for review of participants’ medical records, and for the collection of blood samples to assess insulin resistance index (IRI), serum uric acid (sUA), serum creatinine (SCr), estimated glomerular filtration rate (eGFR), triglycerides (TG), total cholesterol (TC), low-density lipoprotein cholesterol (LDL-C), and high-density lipoprotein cholesterol (HDL-C).

### Interventions

#### Explanation for the choice of comparators {6b}

A total of 60 patients who are overweight and obese patients with gout who meet the study criteria will be randomized (1:1) to the moderate-intensity aerobic exercise group and control group.

#### Intervention description {11a}

1. Control group

Participants in the control group will be given general health education, including knowledge about regular exercise, moderate water intake, restriction of alcohol consumption, and high sugar and purine diet intake. Participants will be asked to avoid making changes in their exercise habits during the study. There is no limit to the method of exercise.

(2) Moderate-intensity aerobic exercise group

Aerobic exercise refers to the physical exercise of low to high intensity that depends primarily on the aerobic energy-generating process. “Aerobic” is defined as “relating to, involving, or requiring oxygen,” and refers to the use of oxygen to meet energy demands during exercise via aerobic metabolism adequately. Examples of aerobic exercise include medium- to long-distance running or jogging, swimming, cycling, stair climbing, and walking. On the basis of general health education, moderate-intensity aerobic exercise will be prescribed with METs using a treadmill exercise test system (T2100-ST2; GE Healthcare, Waltham, MA, USA) and an automated sphygmomanometer (SunTech Medical Tango M2; SunTech Medical, Inc., Morrisville, NC, USA). The target HR and exercise speed of patients with gout will be assessed by the HR reserve and Bruce protocol, respectively. The Karvonen method will be used to target an exercise intensity of 60% of HR reserve as follows: HRR= ((HRmax-HRrest) × Intensity (%)) + HRrest, where the maximum HR (HRmax) uses the submaximal HR [85%×(220-age)] during the exercise tests. Exercise tests will be performed according to the Bruce protocol. Therefore, the target HR calculated by the HR reserve method will be used as the target HR of moderate-intensity aerobic exercise. The speed of aerobic exercise corresponding to the MET value of target HR calculated by the HR reserve method will be used as the main exercise program of the test group (Table 1). If participants cannot tolerate running, the mode will be switched to cycling. At the same time, aerobic exercise of reasonable intensity is determined by evaluating subjective physical sensation with the Borg rating of perceived exertion (RPE) scale (Table 2) [14]. For the moderate-intensity aerobic exercise session, which is corresponded to RPE of “12–13,” it is recommended to exercise for 30–40 min/session (excluding warm-up and stretching), 3–5 times/week, and maintain 120–150 min/week. The venue includes sidewalks, parks, gyms, and other appropriate places for exercise.

(3) Trial period

**Table 1:** Energy expenditure levels of running and cycling

Energy expenditure levels	3∼4 METs	5∼6 METs	7∼8 METs	>9 METs
Mode				
Running (km/h)	4.8-6.4	7.2-8.0	8	10
Cycling (km/h)	13	14.5-16.0	19	>21

**Table 2:** Borg rating of perceived exertion scale

RPE	subjective feeling	reference heart rate
6	ease	HRrest
7	very, very light	70 bpm
8		
9	very light	90 bpm
10	fairly light	
11		110 bpm
12	somewhat hard	
13		130 bpm
14		
15	hard	150 bpm
16	very hard	
17		170 bpm
18		
19	very, very hard	195 bpm
20		HRmax

1–12-week: HRR=((HR_max_-HR_rest_)×60% intensity)+HR_rest._ The target HR calculated by the HR reserve method will be used as the target HR of moderate-intensity aerobic exercise. The exercise speed of patients with gout corresponding to the MET value of target HR calculated by the HR reserve method will be used as the main exercise program in the test group.

During the trial, (1) all participants will receive general administration of gout, (2) all participants will be asked to avoid making changes in their dietary habits, (3) participants should keep records of training sessions during the whole intervention, and (4) participants will be asked to avoid performing exercise after drinking or after taking sedatives and analgesics.

### Research procedures

1. Screening

According to the ACR/EULAR criteria and the scoring system for gout presented in 2015, we will adopt the following criteria: patients with gout (in intermittent or chronic phase; there will be no gout flare in the last 2 weeks); patients aging 18–55-years-old; patients with BMI ≥ 24 kg/m^2^.

(2) Enrollment

Participants will be randomly assigned into test and control groups, and participants in both groups will be treated for 12 weeks.

(3) Program

Moderate-intensity aerobic exercise group:

The target HR calculated by the HR reserve method will be used as the target HR in the moderate-intensity aerobic exercise. The exercise speed of patients with gout corresponding to the MET value of target HR calculated by the HR reserve method will be utilized as the main exercise program in the test group. The HR reserve will be calculated as follows: HRR = [(HRmax-HRrest) × 60% Intensity] + HRrest. The exercise will be performed for 30–40 min/session and 3 times/week. The total duration of exercise is 120–150 min/week.

Control group:

No restriction will be applied in terms of the mode of exercise, and participants can select their preferred exercise regimens during the trial. They will be asked to avoid making changes in their exercise habits.

(4) Laboratory indicators

Weight, body fat, waist circumference, hipline, blood pressure, blood glucose, routine blood test, urine routine test, and renal and liver functions will be assessed. The body composition will be determined by BIA.

(5) Follow-up

Follow-up will be conducted at baseline, 6 weeks ± 3 days, and 12 weeks ± 3 days.

(6) Data and specimen collection

Participants’ demographic data will be collected in terms of age, gender, blood pressure, course of the gout, history of medications, etc. Participants will be followed up for three times (baseline, 6 weeks, and 12 weeks after the test) to measure height, weight, body fat, waist circumference, hipline, BMI, blood routine (e.g., sUA), urine routine (including urinary pH), and biochemical indices (blood glucose, serum insulin, triglycerides (TG), total cholesterol (TC), low-density lipoprotein cholesterol (LDL-C), high-density lipoprotein cholesterol (HDL-C), serum creatinine (SCr), and estimated glomerular filtration rate (eGFR)). Predictors of relevant systems will be evaluated after 12 weeks. Biological specimens will be destroyed after testing.

### Criteria for discontinuing or modifying allocated interventions {11b}

#### Criteria for discontinuing

1. Participants withdraw the trial spontaneously.
2. Pregnancy (pregnant participants should withdraw the trial).
3. Adverse events that persuade investigators to urge participants to leave the trial as early as possible.
4. Poor compliance of participants with the research program, and exercise adherence rate is not in the range of 80–120%.
5. Occurrence of serious adverse reactions related to exercise intervention.
6. In this study, major mistakes are detected in the clinical research program, making it difficult to evaluate the efficacy of the intervention. In addition, a significant deviation is observed in the implementation of a well-designed program.

### Strategies to improve adherence to interventions {11c}

To increase participants’ compliance in the test group, it will be essential to record details of exercise (including date, start/end time, and rate) and take screenshots to retain data by the mobile phone application. Participants’ details of exercise in the control group will be recorded according to their exercise habits. Data from both groups will be forwarded to research team every week. Weekly telephone follow-up will be performed to strengthen participants’ compliance and to provide a recommendation sticking to an exercise program.

### Relevant concomitant care permitted or prohibited during the trial {11d}

*This* trial will not impose *special requirements for* care and interventions.

### Provisions for post-trial care {30}

When the participants complete the trial, they will receive standardized treatment based on the guideline.

### Outcomes {12}

#### Primary outcome

Primary outcome will include the number of patients whose body fat is reduced after 12 weeks.

#### Secondary outcomes

Secondary outcomes will include changes in BMI, waist-to-hip ratio (WHR), TG, TC, LDL-C, HDL-C, insulin resistance index (IRI), hepatic steatosis, sUA, SCr, eGFR, and adverse effects after 12 weeks. Adverse events will be monitored during the 12 weeks of study, including the rate of gout flare. Any adverse reactions that occur during the study will be recorded in the “Adverse Reaction Table,” and participants will be followed up until symptoms disappear or indicators return to normal. In the serious adverse events, necessary measures will be taken immediately to ensure participants’ safety.

### Participant timeline {13}

**Fig. 2:**
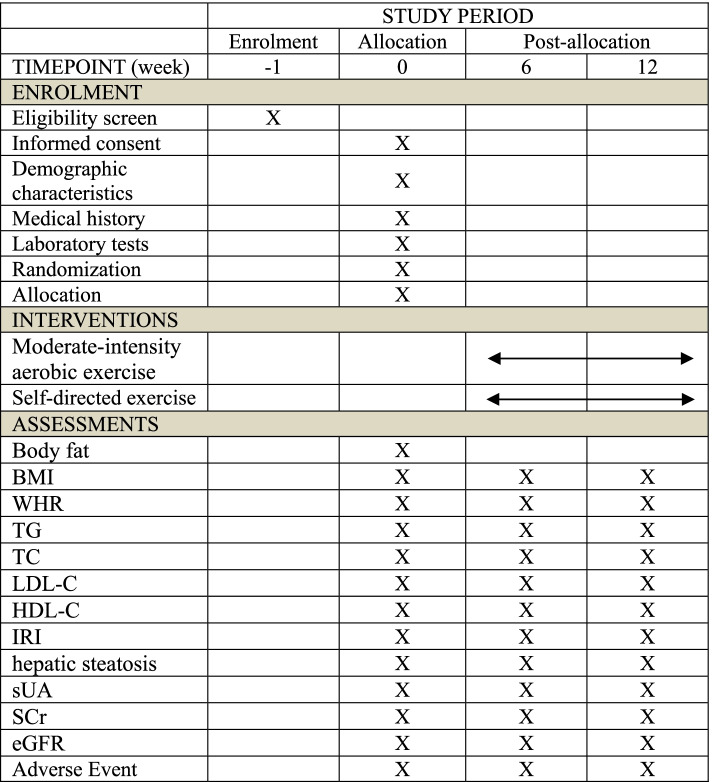
SPIRIT figure of enrolment, interventions, and assessments

The participant timeline in shown in Fig. 2.

### Sample size {14}

This is a differential test, and the primary outcome is the number of patients whose body fat is reduced after 12 weeks of exercise intervention. The sample size is 50 with involvement of 25 patients in the intervention and control groups. Considering a 15% dropout rate, the final sample size will be 30 patients per each group, and the two groups will totally include 60 cases.

### Recruitment {15}

Participants will be recruited from Shenzhen Traditional Chinese Medicine Hospital, People’s Hospital of Longhua District Shenzhen, and the Second Affiliated Hospital of Guizhou University of Traditional Chinese Medicine between September 2022 and January 2024. A total of 60 adult patients with gout (no gout flares can be detected in the last 2 weeks) from outpatient and ward screening programs aging 18–55 years old will be invited to attend in the bioelectrical impedance analysis (BIA). Once a patient is confirmed to be eligible, a physician will review the study procedures and requirements with the patient and ask his/her commitment to the study and acceptance of randomization. Written informed consent will be obtained from each participant, and baseline evaluation will be performed, which will be immediately scheduled by the physician assistant.

### Assignment of interventions: allocation

#### Sequence generation {16a}

Statisticians will adopt the PROC PLAN process of the SAS 9.4 statistical software to generate a random list by blocked randomization of variable block lengths.

#### Concealment mechanism {16b}

In the mechanism of implementing the allocation sequence, Dr. Zhiying Zhan generated the allocation sequence; sequentially numbered, opaque, sealed envelopes can be reproduced when needed.

#### Implementation {16c}

Dr. Zhiying Zhan (Fujian Medical University School of Public Health) generated the allocation sequence; doctors will enroll participants, and the nurse will assign participants to interventions.

### Assignment of interventions: blinding

#### Who will be blinded {17a}

Although this is an open-label trial, the research assistants who collect data will be blinded to participants’ intervention assignment. In addition, adjudicators for end-points will not be aware of study-group assignments.

#### Procedure for unblinding if needed {17b}

The design is open label with only outcome assessors being blinded, so unblinding will not occur.

### Data collection and management

#### Plans for assessment and collection of outcomes {18a}

Participants’ demographic data will be collected in terms of age, gender, blood pressure, course of the gout, history of medications, etc. Participants will be followed up for three times (baseline, 6 weeks, and 12 weeks after the test) to measure height, weight, body fat, waist circumference, hipline, BMI, blood routine (e.g., sUA), urine routine (including urinary pH), and biochemical indices (blood glucose, serum insulin, triglycerides (TG), total cholesterol (TC), low-density lipoprotein cholesterol (LDL-C), high-density lipoprotein cholesterol (HDL-C), serum creatinine (SCr), and estimated glomerular filtration rate (eGFR)). Predictors of relevant systems will be evaluated after 12 weeks. Biological specimens will be destroyed after testing.

The case report form (CRF) is used to assess and collect outcome and baseline. In addition, the clinical trial database is constructed by a designated data manager who is responsible for the regular database management and maintenance. All data will be imported into the clinical trial database by two research assistants.

#### Plans to promote participant retention and complete follow-up {18b}

Follow-up will be conducted at baseline, 6 weeks ± 3 days, and 12 weeks ± 3 days. Any missing or incorrect data will be detected by software system. In such case, the original CRFS will be checked to correct or complete every piece of data.

#### Data management {19}

The CRF for each participant should be filled out in a timely manner. All data will be double-entered by a researcher, including screening assessments, questionnaires, physical examinations, and laboratory examinations. Original documents and CRFs will be stored in the study office. The clinical trial database is structured by the appointed data manager who is responsible for the regular database management and maintenance. The data of this clinical trial is managed by Dr. Zhiying Zhan (Public Health School of Fujian Medical University, Fuzhou, China), ensuring the authenticity, integrity, and privacy of clinical data during the trial.

#### Confidentiality {27}

The confidentiality measures are as follows. At the time of recruitment, participants were contacted directly by the investigator to prevent their information from being known to third-party. The data involved in the study should be paid attention to protect the privacy of the subjects. For example, the cover of the CRF and the information page should be the initials of the participants. The participant’s signature in the informed consent form should be kept separately from other data. In publishing the results of the study, we report them using aggregate data from the population rather than individual data from the participants. When describing the special situation of an individual, personal information that can identify the subject should not be presented and can be coded instead.

#### Plans for collection, laboratory evaluation, and storage of biological specimens for genetic or molecular analysis in this trial/future use {33}

The case report form will be used to assess and collect outcomes and baseline. In addition, all data will be imported into the clinical trial database by 2 research assistants. The investigator will be responsible for maintaining accurate, complete, and up-to-date records for each subject. The investigator will also be responsible for maintaining any source documentation related to the study, including any films, tracings, or computer discs. All biological specimens will be destroyed after the analysis.

### Statistical methods

#### Statistical methods for primary and secondary outcomes {20a}

Statistical analysis will be carried out using SAS 9.4 statistical software, and all data will be expressed as mean ± standard deviation (SD) or percentage (%). Differences in demographic data, anthropometric measurement, blood biochemical parameters, and health-related physical fitness components between baseline and week 12 groups will be analyzed using the paired *t*-test. One-way analysis of variance (ANOVA) will be used to compare the mean values of normally distributed variables between MIAEG and GC. Statistical analysis was carried out using SPSS (version 26). A two-tailed significance level of 0.05 was used for all tests. *P* < 0.05 is considered statistically significant.

#### Interim analyses {21b}

The Trial Steering Committee will have access to these interim results and make the final decision to terminate the trial.

#### Methods for additional analyses (e.g., subgroup analyses) {20b}

The hybrid control will use multivariate logistic regression, estimating the odds ratio (OR) and 95% confidence interval (CI). Clinically significant variables in the univariate analysis will be included in the multivariate model. The goodness of fit will be evaluated by Hosmer–Lemeshow test.

#### Methods in analysis to handle protocol non-adherence and any statistical methods to handle missing data {20c}

Study populations include the intent-to-treat (ITT) analysis set defined as all randomized patients, and the per-protocol (PP) analysis set defined as all patients in the ITT population without any major protocol deviations. Multiple imputation is used to manage missing values.

#### Plans to give access to the full protocol, participant level-data, and statistical code {31c}

Data are available upon reasonable request. For inquiries about data sharing, please send a request to sailing1980@126.com.

### Oversight and monitoring

#### Composition of the coordinating center and trial steering committee {5d}

The trial steering committee consists of three members, two senior rheumatologists and a statistician, who will oversee the trial. The committee is independent of the research team and there is no conflict of interest. Information on the composition, roles and responsibilities of group providing day-to-day support to the trial is provided below. Three rheumatologist collect clinical data.

#### Composition of the data monitoring committee, its role and reporting structure {21a}

The data monitoring committee consists of three members, two senior rheumatologists and a statistician, who will ensure the safety and quality of data. The committee is independent of the research team and there is no conflict of interest. They will provide regular supervision, hold monthly meetings, and organize a field trip at least once to ensure the trial is carried out smoothly and ethically. Also, a supervisor will ensure the authenticity and integrity of the data. During the visit, they will interview the investigators, check the original research documents and subject registration, and confirm whether the clinical centers comply with the research protocol. Any non-compliance with the agreement will be fully recorded using a violation report form.

#### Adverse event reporting and harms {22}

In this study, if a lot of exercise leads to excessive production of lactic acid, which can easily lead to the formation of urate crystals and induce gout flares. Ankle sprain, injury, and other conditions may occur during exercise. For obese or overweight people with gout, vigorous anaerobic exercise should be avoided, and stretching should be carried out before exercise to prevent injury.

#### Frequency and plans for auditing trial conduct {23}

We will audit the trial conduct every month. The process will be independent from investigators and the sponsor.

#### Plans for communicating important protocol amendments to relevant parties (e.g., trial participants, ethical committees) {25}

If investigator any decision to amend the protocol has to be made, a written application need to be submitted to the Institutional Medical Ethics Committee, and the investigator will be notified in writing after approval. The protocol will be updated immediately in the system. Participants also have the right to be informed. In addition, the investigator needs to apply to the clinical trial registry for protocol change approval.

#### Dissemination plans {31a}

The results will be released after the data analysis. The primary findings will be presented via publications, including announcement and articles in the clinical trial registry. The datasets analyzed in the current study will be available from the corresponding author upon request.

## Discussion

To date, the treatment strategy for gout has shifted from medical therapy alone to comprehensive management of the disease, and there is increasing interest in lifestyle interventions as cost-effective treatments for gout. The increase of sUA level is canonically considered as an important biochemical basis of gout, and obesity can increase the sUA level [15]. It was confirmed that exercise is a promising intervention for patients with gout [10]. It was found that the long-term moderate-intensity aerobic exercise remarkably reduced the incidence of gout, improved the BMI and whole-body metabolism, lowered UA levels, and decreased the incidence of complications. Vigorous exercise, however, significantly increased UA levels [16]. It is necessary to recommend moderate-intensity aerobic exercise for patients with gout, while research on the status of exercise training for gout patients is still lacking, and no specific exercise training programs for such patients have been extensively organized. The effect and optimal frequency of exercise for improving the status of overweight and obese patients with gout have not yet been determined. To develop an individualized exercise program for patients with gout, we designed a 12-week randomized controlled trial and evaluated the effects of individualized aerobic exercise program on patients with gout.

Participants in this trial have BMI ≥ 24 kg/m^2^ and heavy weight-bearing. When formulating exercise prescriptions, jogging, cycling, and other forms of exercise with less joint damage can be selected, while the risk of exercise load and joint injury should not be increased.

Exercise capacity of gout is reduced compare with that in normal. According to the patient’s condition, it is essential to select the appropriate form of exercise for the patient, adhere to the completion of the corresponding length of exercise type, and reduce the patient’s fear of exercise and obstacles. Patients are mainly accompanied by pain of lower limbs after gout flares. The persistence and motivation of exercise may also be affected after remission. Additionally, the sample size of this trial is small, which may lead to bias. A trial with larger sample size and long-term follow-up is required to find out a more appropriate exercise program for gout patients.

In the present trial, we aim to identify an optimal level of exercise intensity for gout patients. The results may assist such patients with a personalized scientific exercise program based on the disease status and motor abilities, so that patients are prone to exercise under the condition of low risk and achieve the greatest benefits. Therefore, the optimal frequency, intensity, and mode of exercise for improving gout need to be tested in the future clinical studies.

## Trial status

The revised version of V20220408 on April 8, 2022, has been approved by the Institutional Medical Ethics Committee of Shenzhen Traditional Chinese Medicine Hospital, People’s Hospital of Longhua District Shenzhen, and the Second Affiliated Hospital of Guizhou University of Traditional Chinese Medicine. The recruitment will begin on September, 2022, and this study will complete on May, 2024.

## Data Availability

Any data required to support the protocol can be supplied on request.

## Abbreviations

RCT: Randomized controlled trial
BMI: Body mass index
ACR: American College of Rheumatology
EULAR: European League Against Rheumatism
CG: Control group
MIAEG: Moderate-intensity aerobic exercise group
HRR: Heart rate reserve
sUA: Serum uric acid
OR: Odds ratio
RPE: Rating of perceived exertion
BIA: Bioelectrical impedance analysis
WHR: Waist-to-hip ratio
IRI: Insulin resistance index
SCr: Serum creatinine
CRF: Case report form
NF-κB: Nuclear factor-κB
TLR2: Toll-like receptor 2
CXCL1: Chemokine ligand 1
METs: Metabolic equivalent
TET: Treadmill exercise test
eGFR: Estimated glomerular filtration rate
TG: Triglycerides
TC: Total cholesterol
HDL-C: High-density lipoprotein cholesterol
LDL-C: Low-density lipoprotein cholesterol
SD: Standard deviation
ANOVA: Analysis of variance
CI: Confidence interval
ITT: Intent-to-treat
PP: Per-protocol
